# A Multicenter Evaluation of Diagnostic Tools to Define Endpoints for Programs to Eliminate Bancroftian Filariasis

**DOI:** 10.1371/journal.pntd.0001479

**Published:** 2012-01-17

**Authors:** Katherine Gass, Madsen V. E. Beau de Rochars, Daniel Boakye, Mark Bradley, Peter U. Fischer, John Gyapong, Makoto Itoh, Nese Ituaso-Conway, Hayley Joseph, Dominique Kyelem, Sandra J. Laney, Anne-Marie Legrand, Tilaka S. Liyanage, Wayne Melrose, Khalfan Mohammed, Nils Pilotte, Eric A. Ottesen, Catherine Plichart, Kapa Ramaiah, Ramakrishna U. Rao, Jeffrey Talbot, Gary J. Weil, Steven A. Williams, Kimberly Y. Won, Patrick Lammie

**Affiliations:** 1 Lymphatic Filariasis Support Center, The Task Force for Global Health, Decatur, Georgia, United States of America; 2 Hopital Ste. Croix, Leogane, Haiti; 3 Noguchi Memorial Institute for Medical Research, University of Ghana, Legon-Accra, Ghana; 4 Global Community Partnerships, GlaxoSmithKline, London, United Kingdom; 5 Washington University School of Medicine, St. Louis, Missouri, United States of America; 6 Research and Development Division, Ghana Health Service, Accra, Ghana; 7 Department of Parasitology, Aichi Medical University School of Medicine, Nagakute, Aichi-ken, Japan; 8 National Program for Elimination of Lymphatic Filariasis, Ministry of Health, Funafuti, Tuvalu; 9 Lymphatic Filariasis Support Centre, School of Public Health, Tropical Medicine and Rehabilitation Sciences, James Cook University, Townsville, Queensland, Australia; 10 Department of Biological Sciences, Smith College, Northampton, Massachusetts, United States of America; 11 Institut Louis Malardé, Papeete, Tahiti, French Polynesia; 12 Anti-Filariasis Campaign, Sri Lanka Ministry of Health and Nutrition, Colombo, Sri Lanka; 13 Neglected Tropical Disease Control Program, Ministry of Health, Zanzibar, United Republic of Tanzania; 14 Vector Control Research Centre, Indian Council of Medical Research, Pondicherry, India; 15 Division of Parasitic Diseases and Malaria, Centers for Disease Control and Prevention, Atlanta, Georgia, United States of America; McGill University, Canada

## Abstract

Successful mass drug administration (MDA) campaigns have brought several countries near the point of Lymphatic Filariasis (LF) elimination. A diagnostic tool is needed to determine when the prevalence levels have decreased to a point that MDA campaigns can be discontinued without the threat of recrudescence. A six-country study was conducted assessing the performance of seven diagnostic tests, including tests for microfilariae (blood smear, PCR), parasite antigen (ICT, Og4C3) and antifilarial antibody (Bm14, PanLF, Urine SXP). One community survey and one school survey were performed in each country. A total of 8,513 people from the six countries participated in the study, 6,443 through community surveys and 2,070 through school surveys. Specimens from these participants were used to conduct 49,585 diagnostic tests. Each test was seen to have both positive and negative attributes, but overall, the ICT test was found to be 76% sensitive at detecting microfilaremia and 93% specific at identifying individuals negative for both microfilariae and antifilarial antibody; the Og4C3 test was 87% sensitive and 95% specific. We conclude, however, that the ICT should be the primary tool recommended for decision-making about stopping MDAs. As a point-of-care diagnostic, the ICT is relatively inexpensive, requires no laboratory equipment, has satisfactory sensitivity and specificity and can be processed in 10 minutes—qualities consistent with programmatic use. Og4C3 provides a satisfactory laboratory-based diagnostic alternative.

## Author Summary

Lymphatic filariasis (LF), a mosquito-borne parasitic disease, is a candidate for elimination largely because of the success of mass drug administration (MDA) campaigns, in which entire at-risk populations are given a once-yearly regimen of single-dose treatment with two medications. As a result, a diagnostic tool is needed to determine when the prevalence of LF has fallen below the threshold for sustained transmission so that MDA programs can be stopped. To determine the best diagnostic tool available, a multi-country study was conducted to assess the performance of seven diagnostic tests on a panel of patient specimens. The selection of the most effective diagnostic test was based on an evaluation of each test's accuracy, technical requirements, programmatic feasibility and reliability, as well as confidence in test performance. This study found advantages and disadvantages to each test. Based on the data and experiences it was determined that the ICT test, a point-of-care rapid card test, is the preferred diagnostic tool for use in defining the end-point of MDA, although the Og4C3 test provides a suitable laboratory-based alternative.

## Introduction

In 2000 the Global Programme to Eliminate Lymphatic Filariasis (GPELF) was launched, providing antifilarial drugs to millions of people through mass drug administration (MDA) programs. During the GPELF's first nine years over 2.6 billion treatments of antifilarial drugs were given to people in 48 countries through MDA programs [1]. The success of the GPELF has led to dramatic reductions of both microfilaremia and antigenemia levels in countries that have completed multiple rounds of MDA [2]; the challenge now is to determine when it is most appropriate to stop MDA [3].

The decision to stop MDA is complicated and a variety of tools have been suggested to guide the decision [4]. The first step is to define the parameter(s) that will be measured and the best diagnostic tool for assessing it. At least seven diagnostic tests are currently available for detecting indicators of LF exposure and infection. Selection of the best diagnostic test for use in stopping-MDA decisions requires consideration of each test's accuracy, technical requirements, programmatic feasibility and reliability [5], as well as confidence in test performance, especially since there is no single gold standard test for LF (see Discussion). Next, following the selection of a preferred diagnostic tool for defining the end-point of MDA, the question of how best to sample the population must be resolved.

In response to these and other challenges, this study was planned to evaluate diagnostic tools to assess MDA program success by standardizing the tools now available, comparing their effectiveness in demonstrating the interruption of LF transmission, and selecting the most effective for deciding when MDA can be suspended [6]. A large multi-country study was conducted in 2007–2008 to compare the effectiveness of seven available diagnostic tests for detecting evidence of *Wuchereria bancrofti* infection or transmission following multiple rounds of MDA, in settings where infection prevalence was likely to be low. The goal of the study was to select the best diagnostic tool(s) that would allow for definition of program end-points that will maximize the likelihood that LF transmission has been interrupted. Such a tool(s) would be the cornerstone of programmatic decision-making.

## Methods

### Site Selection

Studies were performed in French Polynesia, Ghana, Haiti, Sri Lanka, Zanzibar (United Republic of Tanzania) and Tuvalu, representing a broad diversity of settings in which LF is present. The study sites were believed to have low residual microfilaremia prevalence rates in the range of 0.5–2% following at least five rounds of MDA [7].

### Participant Selection

One community survey and one school survey were performed in each country. Community surveys sampled residents of selected households between the ages of 3 and 80. School surveys were performed in primary schools that serve children in the same villages as those selected for the community surveys. First and fourth year students (approximately 6 and 10 years old, respectively) were selected for inclusion in the school surveys. Children from the school survey were excluded if they had already been included in the community survey. Since the primary objective of this first phase of research was not to assess program end-points in the specific study sites, but rather to compare test effectiveness in the same groups of individuals late in program activities, convenience sampling was used to select both communities and schools. However, selection of participants within each site was conducted randomly whenever possible.

### Standard Operating Procedures

A workshop with all the investigators was held in Atlanta, GA to establish the study protocols and Standard Operating Procedures (SOP) [7] prior to the start of the study. For each country, a team of experienced investigators traveled to the study site to train the local team on data collection methods and laboratory procedures in accordance with the SOP.

### Demographic Data Collection

All information on the participants was collected using handheld personal digital assistants (PDA) (Dell Axim X50 or X51) that eliminated the need for paper records. Unique identifiers were printed on labels which provided visual identification of the number as well as barcodes acquired by a Bluetooth® scanner (CHS 7p v.1, Socket Mobile) to facilitate specimen management. The PDAs were equipped with GPS devices (GlobalSat, City of Industry, CA, USA) and GPS coordinates were captured at each house and school visited. A questionnaire was administered to collect demographic information that included age, gender, bednet use, self-reported filarial disease status and compliance with the most recent MDA. Multiple teams could register households at the same time, and data collected could be synchronized in the field to create one master database. Each night all data were uploaded to a field laptop and a backup of the data was created on an external drive. Data were electronically transmitted in the form of encrypted excel files to the central analysis database at the Task Force for Global Health (Atlanta, GA).

### Blood and Urine Collection

All field sample collections and field and laboratory tests were conducted according to the SOP. Blood and urine samples were collected 6–24 months following the last MDA. The periodicity of *W. bancrofti* required that blood collection in the community surveys be performed during the peak hours of microfilaremia (during daytime hours for French Polynesia and Tuvalu and between 10 pm and 2 am in the remaining countries where the parasite was nocturnally periodic). In the areas with nocturnal periodicity, collection teams had the option of registering households during the day or night. Teams that registered households during the day later returned in the evening to take the blood samples. Approximately 0.3–0.4 ml of blood was collected by finger prick from each participant into an EDTA coated blood collection tube and stored in coolers overnight before assays were performed the next day in the field laboratory. Up to six diagnostic assays were performed (with the exception of Ghana, which conducted up to five assays). Three of the assays were conducted in the field laboratory: blood smear (MF), ICT (Immunochromatographic test, Binax, Scarborough, ME), and the PanLF Rapid (MBDr, Selangor, Malaysia). The one exception to this was French Polynesia where the blood smear, ICT and PanLF assays were processed at the Institut Louis Malardé. The Bm14 antibody detection and Og4C3 antigen detection assays were conducted in five reference laboratories (see Table 1) and the PCR (Polymerase Chain Reaction) tests were conducted at Smith College in Northampton, MA, USA.

**Table 1:** Laboratory locations of diagnostic tests.

	Bm14	PanLF	Urine SXP	ICT	Og4C3	Blood Smear	PCR*
French Polynesia	ILM	ILM	Aichi	ILM	ILM	ILM	Smith
Ghana	Noguchi	–	–	Field lab	Noguchi	Field lab	Smith
Haiti	CDC	Field lab	Aichi	Field lab	CDC	Field lab	Smith
Sri Lanka	Wash U.	Field lab	–	Field lab	Wash U.	Field lab	Wash U.
Tuvalu	Wash U.	Field lab	Aichi	Field lab	Smith	Field lab	Smith
Zanzibar	Smith	Field lab	Aichi	Field lab	Smith	Field lab	Smith

Aichi = Aichi Medical University (Japan).

CDC = Centers for Disease Control and Prevention (USA).

Field Lab = in-country laboratory created, or in use, by field team.

ILM = Institut Louis Malarde (French Polynesia).

Noguchi = Noguchi Memorial Institute for Medical Research (Ghana).

Smith = Smith College (USA).

Wash U = Washington University in St. Louis, Missouri (USA).

*Based on 10 µl blood specimen.

For school participants, four diagnostic assays were performed: two conducted on site (ICT and PanLF) and two conducted in reference laboratories (Bm14 and Og4C3). Because microfilaremia levels were not assessed in the school surveys, blood collection occurred during the day at the time of registration.

Urine cups were labeled and distributed at the time of enrollment, and each participant was asked to provide a urine sample (with the exception of those in Ghana and Sri Lanka). In the field laboratory, approximately 5 ml of urine was transferred into a smaller vial and sodium azide (0.1%) was added as a preservative [8]. Urine vials were shipped to Aichi Medical University (Nagoya, Japan) for anti-filarial antibody testing using the *W. bancrofti* SXP recombinant antigen. Table 2 summarizes the tests by: survey, specimen, test type, and target detected.

**Table 2:** Diagnostic test characteristics.

Test Name	Surveys Used	Specimen Type	Test Type	Target Detected
**Bm14**	Community & School	Bloodspot	ELISA	Antifilarial antibody
**PanLF**	Community & School	Blood	Rapid cassette test	Antifilarial antibody
**Urine SXP**	Community & School	Urine	ELISA	Antifilarial antibody
**ICT**	Community & School	Blood	Rapid card test	Filarial-antigen
**Og4C3**	Community & School	Bloodspot	ELISA	Filarial-antigen
**Blood Smear**	Community	60 µl Blood	Blood film	Microfilariae
**PCR** *	Community	10 µl Bloodspot	qPCR	Microfilariae

*Based on 10 µl blood specimen.

### Field Tests

Blood films were used to determine MF levels in the communities. Sixty microliters of blood was streaked onto a glass slide (3 lines×20 µl), stained with Giemsa and read in the field laboratories. Filarial-antigen status was determined by ICT (Binax, Scarborough, ME, USA). EDTA anti-coagulated blood was used and the test was performed according to manufacturer's instructions. Antigen positive individuals were offered treatment with albendazole plus DEC or ivermectin. Anti-filarial antibody status was determined using the PanLF Rapid (MBDr, Selangor, Malaysia) cartridge test. EDTA anti-coagulated blood (35 µl) was placed on the sample pad and the test was performed according to manufacturer's instructions. The remaining blood was spotted onto two filter paper disks (TropBio, Townsville, Australia) (60 µl per disk), dried and stored until shipped to participating laboratories for further testing.

Both the ICT and PanLF tests were conducted at the schools and blood was spotted onto filter paper disks. All field test results were entered into the PDA immediately and subsequently uploaded to the field laptop each night.

### Laboratory Tests

Three laboratory assays were performed on the specimens, all of which were previously validated against non-endemic samples. One bloodspot (10 µl) was used for an enzyme linked immunosorbent assay (ELISA) to determine anti-filarial antibody reactivity to the recombinant antigen Bm14 (Cellabs, Sydney, Australia). Bloodspots were eluted overnight at 4°C and processed the following day according to the agreed SOP. Three dried bloodspots (3×10 µl) were used to measure quantitative filarial antigen levels by the Og4C3 ELISA (TropBio, Townsville, Australia). Bloodspots were eluted overnight at 4°C and boiled the next day. Boiled samples were centrifuged and supernatants were incubated overnight on a 96-well microtiter plate pre-coated with an Og4C3 monoclonal capture antibody. Plates were processed the next day. One bloodspot (10 µl) was used for PCR to detect the presence of parasite DNA. Bloodspots were pooled into groups of 10 individuals for initial testing. DNA was extracted using the QIAGEN DNeasy kit (Valencia, CA, USA) and analyzed by real-time PCR (qPCR) [9]. If a pool was positive, each sample that comprised the positive pool was tested individually using an additional 10 µl bloodspot. Results for all laboratory tests were entered into a standardized Microsoft Excel® spreadsheet and sent electronically to the Task Force for Global Health to be entered into the analysis database.

### Ethics Statement

The research proposal was submitted by the principal investigators of each participating country to the local review board, or in certain cases an outside review board, as deemed most appropriate. All proposals were accepted by the respective review boards before research took place. The US-based laboratories analyzing results received an exemption from the IRBs, since all specimens and results were de-linked from personal identifiers. All subjects provided informed consent to participate in the study. More detailed information regarding the IRB institution for each country and the method for obtaining participant consent are described below.

In French Polynesia, the Ethics Committee approved the French Polynesian study protocol and work. A consent form was read to all a subjects and written agreement of consent was required from subjects in order to participate in the study. Assent was obtained from children and a written consent was required from their parent or guardian. In addition to obtaining written consent from participants, interviewers documented receipt of consent for all participants using handheld PDA devices. For Ghana, the Noguchi Memorial Institute for Medical Research's Institutional Review Board approved the study protocol and work. Informed written consents were obtained from all individuals 18 years of age and above. For individuals aged 6–17 years informed assent was sought from all individuals, in addition to written consent of the parent or responsible adult. In addition to obtaining written consent from participants, interviewers documented receipt of consent for all participants using handheld PDA devices. The procedure was explained to all children 3–5 years of age, in addition to written consent of the parent or designated guardian. In Haiti the Centers for Disease Control IRB committee approved an amendment to a previously approved study protocol. Informed consent was obtained from each participant. The CDC IRB granted the team the right to obtain oral consent (assent for children of age 6 years or younger and consent of their parents) because most participants were unable to read and the research presented no more than minimal risk of harm to the subjects. Interviewers documented receipt of verbal consent for all participants using handheld PDA devices. In Sri Lanka both the Washington University IRB and the Sri Lanka Ministry of Health approved the study protocol and work. Both institutions considered the survey to be public health practice (evaluation of the national LF elimination program) and as a result did not require formal IRB submission; waiver letters were obtained. Field teams used consent scripts and obtained verbal consent (assent from children). Participation by children required consent from at least one parent plus assent from the child. The Washington University IRB and Sri Lanka Ministry of Health both approved the collection of verbal consent for the survey because the research was deemed to present no more than minimal risk of harm to the subjects. Interviewers documented receipt of verbal consent for all participants using handheld PDA devices. For Tuvalu the human research ethics committee at James Cook University approved the protocol and study. The ethical review committee at James Cook University granted the right to obtain verbal consent, as opposed to written consent, for this study, as the study was considered to present minimal risk of harm to the subjects. Assent was obtained from children, along with verbal consent from their parent or guardian. Interviewers documented receipt of verbal consent for all participants using handheld PDA devices. Finally, the Ethical Review Committee in Zanzibar (Zanzibar Health/Medical Task Force) approved the Zanzibar study protocol and work. For the community all participants were given consent forms to sign while for the school children parents/guardians of the children were informed of the study through School Committee meetings and an informed consent letter was handed over to them to be signed. In addition to obtaining written consent from participants, interviewers documented receipt of consent for all participants using handheld PDA devices.

### Analyses

All data were compiled and managed using SQL server (2005, Microsoft Corporation®) and imported to SAS® v.9.2 (Statistical Analysis System; North Carolina) for analyses. Unless otherwise stated, all statistically significant associations were determined by setting the probability of a Type I error at 5% (α = 0.05). Univariate analyses of country, age, and gender were calculated for all specimens with results reading “positive”, “negative”, and “indeterminate” (Tables 2 and Table 3). For all remaining analyses results were limited to specimens testing “positive” or “negative.”

**Table 3:** Demographic information by country and survey location.

Location	Measure	French Polynesia	Ghana	Haiti	Sri Lanka	Tuvalu	Zanzibar	All Countries
Community	Age (median)	33	17	17	26	39	24	25
	Age (IQR)	16–48	10–40	10–28	13–40	25–50	14–41	13–43
	Percent Male	48	43	41	49	47	39	44
	Total Tested	1018	1107	999	1167	1124	1028	6443
School	Age (median)	7	7	7	7	9	9	7
	Age (IQR)	7–10	6–10	6–10	6–10	7–10	6–10	6–10
	Percent Male	50	49	49	63	48	48	51
	Total Tested	365	359	323	310	357	356	2070
All	Age (median)	20	12	12	19	29	16	17
	Age (IQR)	9–42	8–30	7–23	8–36	10–46	10–36	9–37
	Percent Male	48	44	43	52	47	41	46
	Total Tested	1383	1466	1322	1477	1481	1384	8513

While five of the seven diagnostic tests provided qualitative (positive/negative) results, two provided quantitative results (Og4C3 and Bm14) in the form of unit values. In order to dichotomize these quantitative results, a cut-off value was defined for the Og4C3 and Bm14 tests, independently, such that all results with a unit value greater than the cut-off were considered “positive.” Receiver Operating Characteristic (ROC) curves were used to determine the best cut-off values, by plotting ‘sensitivity’ by ‘1-specificity’ at various signal to cut-off ratios using SAS®. ROC analysis requires identifying clearly positive and negative specimens whose assay values can be applied to the analysis, but since there is no true ‘gold standard’ for defining LF infection, operational criteria based on multiple tests were used to define these groups.

This manuscript followed the Standards for the Reporting of Diagnostic accuracy studies (STARD) (Checklist S1).

## Results

A total of 8513 people from the six countries participated in the study; 6443 through the community surveys and the remaining 2070 through the school surveys (Table 3). Specimens from these participants were used to conduct 47,110 diagnostic tests (Table 4). Of the 47,110 tests performed, 7481 test results (15.9%) were excluded from the subsequent analyses due to invalid or indeterminate test results (Table 5). Among the excluded results were all of the Bm14 tests for Sri Lanka, Tuvalu and Zanzibar (4006 tests) due to changes in the performance of the commercially manufactured kits. In addition to the Bm14, all of the PanLF and blood smear results from Zanzibar (a total of 2,329 tests) were excluded due to technical uncertainties affecting the quality of the results. Diagrams describing the process by which participant specimens were tested, excluded and classified for each of the antibody, antigen and microfilariae tests are available in the supplementary Texts S1, S2, and S3.

**Table 4:** Specimens and tests performed by country of origin.

Test Name	French Polynesia	Ghana	Haiti	Sri Lanka	Tuvalu	Zanzibar	All Countries
**PanLF**	1372	0	1269	1399	1448	1377	6865
**Bm14**	1329	1159	1214	1463	1245	1298	7708
**Urine SXP**	1268	0	1285	0	955	1366	4874
**ICT**	1359	1372	1266	1449	1455	1316	8217
**Og4C3**	1355	1355	1179	1432	1333	1126	7780
**PCR** *	1005	972	893	1161	1063	886	5980
**Blood Smear**	713	1081	882	1043	1015	952	5686
**TOTAL**	8401	5939	7988	7947	8514	8321	47110

*Based on 10 µl blood specimen.

**Table 5:** Invalid or indeterminate test results by country (excluded from remaining analyses).

Test	French Polynesia	Ghana	Haiti	Sri Lanka	Tuvalu	Zanzibar	All Countries
**PanLF**	66	–	48	435	382	1377	2308
**Bm14**	0	0	0	1463	1245	1298	4006
**Urine SXP**	0	–	0	–	0	0	0
**ICT**	25	119	33	1	7	30	215
**Og4C3**	0	0	0	0	0	0	0
**PCR** *	0	0	0	0	0	0	0
**Blood Smear**	0	0	0	0	0	952	952
**TOTAL**	91	119	81	1899	1634	4009	7481

Note: These test results make up 15.9% of the total (47,110) results.

*Based on 10 µl blood specimen.

ROC curves were used to determine the unit value cut-point to distinguish ‘positive’ and ‘negative’ results for the Og4C3 and Bm14 tests. For the Og4C3 antigen assessment true positives were defined as those individuals with positive specimens for either the blood smear (MF) test or PCR (parasite DNA). True negatives were defined as individuals with negative blood smears and PCR results (both negative or one negative and the other not assessed), plus a negative by ICT and a Bm14 antibody value <18 units. The resulting ROC plots provided strong evidence that the cut-off for defining an Og4C3 positive result should be 34 units.

Determining the cut-point for the antibody assay Bm14, using the ROC, was more problematic. An antibody response is the first identifiable marker following *exposure* to filarial infection, it is therefore impossible to define true-positive *infections* by the presence of antibody. Assay sensitivity can be determined with respect to microfilaremia or antigenemia; however, specificity cannot be conceptually assessed (see Discussion). Indeed ROC analysis for the Bm14 cut-off proved to be inconclusive. Instead it was decided that positivity and negativity be discriminated on the basis of Optical Density values, based on a standard curve run for each test plate [7]. Therefore, the value of 64 units was used as the cut-off, which follows the manufacturer's recommendations and is consistent with the available ROC findings.

As shown in Table 6, 22.8% of participants' specimens had valid results for the full battery of seven tests while almost two thirds of participant specimens had valid results for five or more tests. Bm14 had the highest prevalence of positive results, with country-specific prevalence reaching 53.1% in Haiti (Table 7). The PanLF antibody and urine SXP antibody tests had the second and third highest positivity, with the highest prevalence found in Haiti (41.5%, Table 7) and French Polynesia (22.5%, Table 7), respectively. Across all countries, 17.5% of specimens were positive by PanLF and 20.5% by urine SXP (Table 8). At the country-level, antigen positivity ranged from around 0.5% in Sri Lanka to over 21.2% in Haiti (Table 7), while overall approximately 9% were positive by ICT and 8% by Og4C3 (Table 8). The tests with the least number of positive results were PCR and blood smear, with approximately 1.5% of specimens testing positive overall, though again positivity varied at the country-level.

**Table 6:** Number of *valid* * tests performed on participants specimens.

No. of tests performed	No. of participants	%	Cumulative %
0	2	0	0
1	59	0.7	0.7
2	257	3.0	3.7
3	605	7.1	10.8
4	1980	23.3	34.1
5	2270	26.7	60.8
6	1397	16.4	77.2
7	1943	22.8	100.0

*Note: This does not include the 7,481 invalid or indeterminate tests.

**Table 7:** Prevalence of positive results by test and country.

	Bm14	PanLF	Urine SXP	ICT	Og4C3	Blood Smear	PCR (10 µl)
**French Polynesia**	46.0%	14.0%	22.5%	9.0%	6.4%	3.8%	2.2%
**Ghana**	9.9%	–	–	6.7%	8.9%	2.1%	0.8%
**Haiti**	53.1%	41.5%	18.5%	21.2%	18.8%	4.3%	4.0%
**Sri Lanka**	–	7.2%	–	3.0%	0.5%	0.4%	0.2%
**Tuvalu**	–	25.2%	20.1%	5.0%	4.9%	0.1%	0.3%
**Zanzibar**	–	–	20.9%	8.1%	8.17%	–	0.8%

**Table 8:** Prevalence of positive results by age group (all countries).

	Bm14		PanLF		Urine SXP		ICT		Og4C3		Blood Smear		PCR*	
Age Group	% Pos.	N	% Pos.	N	% Pos.	N	% Pos.	N	% Pos.	N	% Pos.	N	% Pos.	N
0–5	30.2	199	15.9	270	5.3	227	6.3	384	5.3	356	0.7	310	0.6	313
06–10	31.8	1300	10.9	1770	6.5	1437	4.8	2507	4.8	2396	1.3	599	1.6	681
11–15	31.1	531	15.6	622	19.7	539	8.5	934	6.6	912	1.1	782	0.7	808
16–20	40.1	314	15.7	515	18.9	440	9.6	668	9.4	652	1.7	637	1.1	663
21–30	36.8	394	20.0	747	21.0	581	10.7	986	7.9	963	1.1	955	0.6	973
31–40	46.1	297	22.4	647	26.9	527	11.9	831	10.7	804	1.5	787	1.3	824
41–50	46.0	265	20.5	599	35.0	511	11.2	785	9.3	762	2.7	737	2.2	781
>50	50.3	402	28.7	691	42.8	612	11.3	954	11.2	935	2.4	882	1.9	942
**TOTAL**	**37.0**	**3702**	**17.5**	**5861**	**20.5**	**4874**	**8.6**	**8049**	**7.6**	**7780**	**1.6**	**5686**	**1.3**	**5980**

*Based on 10 µl blood specimen.

Though the overall levels of positivity were similar within targets of detection (antibody, antigen or microfilaremia), at the individual level the tests differed significantly. A comparison of the blood smear and PCR results using McNemar's test, matched on participant, found a significant difference between the two tests (p = 0.024). Likewise, a comparison of the ICT and Og4C3 results found the two antigen tests to be significantly different (p = 0.003). The prevalence of antifilarial antibodies differed significantly (p<0.0001) between Bm14, PanLF, and urine SXP tests. The results from all seven diagnostic tests indicated a significant age-prevalence trend of increasing positivity with age (p<0.0001) (Table 8). Of the diagnostic tests, the Bm14 and PanLF were found to be the most reactive in the youngest age groups. In the school studies, which focused on a comparison of 5–7 and 9–11 year olds, there were no significant differences in test results between the two age groups, and the results were subsequently pooled.

The test concordance tables (Tables 9, 10, 11,12) record the pair-wise comparisons of test results within the school and community surveys. The resulting estimates can be considered the pair-wise sensitivity of the test. In the school survey, Og4C3 picked up 57% of the ICT positive results, whereas ICT picked up 51% of the Og4C3 positive results (Table 9). Among the antibody tests, Bm14 identified 90% of the positive PanLF results, whereas PanLF only identified 41% of the Bm14 results. These differences reflect the greater sensitivity of the ELISAs compared to the rapid tests. The urine SXP tests consistently identified about a quarter of the positive results from the remaining four tests.

**Table 9:** Positive-to-positive concordance in school survey.

COMPARISON TEST (Numerator)					
INDEX TEST (Denominator)	BM14	PANLFBC	URSXP	ICT	OG4C3
**BM14**		120/292 **(41%)**	38/283 **(13%)**	45/311 **(14%)**	53/310 **(17%)**
**PanLF**	120/133 **(90%)**		33/136**(24%)**	44/145 **(30%)**	54/138 **(39%)**
**Urine SXP**	38/42 **(90%)**	33/44 **(75%)**		16/69 **(23%)**	18/64 **(28%)**
**ICT**	45/61 **(73%)**	44/63 **(69%)**	16/66 **(24%)**		42/74 **(57%)**
**Og4C3**	53/63 **(84%)**	54/64 **(84%)**	18/77 **(23%)**	42/82 **(51%)**	

Note: Fractions represent the number of positive results for each test (numerator) out of those that were positive by the index test (denominator). The results are of the form: proportion (%). The number of positive index tests (denominator) changes by column because it only includes specimens with valid results by the comparison test (numerator).

**Table 10:** Positive-to-positive concordance in community survey.

	COMPARISON TEST (Numerator)						
INDEX TEST (Denominator)	BM14	PANLFBC	URSXP	ICT	OG4C3	BLOOD SMEAR	PCR*
**BM14**		463/903 **(51%)**	386/905 **(43%)**	231/1015 **(23%)**	237/1033 **(22%)**	54/904 **(6%)**	55/1044 **(5%)**
**PanLF**	463/516 **(90%)**		370/714 **(52%)**	216/869 **(25%)**	220/841 **(26%)**	47/783 **(6%)**	47/854 **(5%)**
**Urine SXP**	386/428 **(90%)**	370/582 **(64%)**		181/878 **(21%)**	193/829 **(23%)**	36/572 **(6%)**	36/853 **(4%)**
**ICT**	231/357 **(65%)**	216/384 **(56%)**	181/455 **(40%)**		299/560 **(53%)**	70/468 **(15%)**	60/571 **(11%)**
**Og4C3**	237/323 **(73%)**	220/292 **(75%)**	193/367 **(53%)**	299/485 **(62%)**		76/397 **(19%)**	68/503 **(14%)**
**Blood Smear**	54/75 **(72%)**	47/65 **(72%)**	36/64 **(56%)**	70/88 **(80%)**	76/87 **(87%)**		52/85 **(61%)**
**PCR** *	55/62 **(89%)**	47/60 **(78%)**	36/66 **(55%)**	60/77 **(78%)**	68/75 **(91%)**	52/69 **(75%)**	

Note: Fractions represent the number of positive results for each test (numerator) out of those that were positive by the index test (denominator). The results are of the form: proportion (%). The number of positive index tests (denominator) changes by column because it only includes specimens with valid results by the comparison test (numerator).

*Based on 10 µl blood specimen.

**Table 11:** Negative-to-negative test concordance in school survey.

	COMPARISON TEST (Numerator)				
INDEX TEST (Denominator)	BM14	PANLFBC	URSXP	ICT	OG4C3
**BM14**		334/347 **(96%)**	338/342 **(98%)**	628/644 **(98%)**	645/655 **(98%)**
**PanLF**	334/506 **(66%)**		566/577 **(98%)**	905/924 **(98%)**	880/890 **(98%)**
**Urine SXP**	338/583 **(57%)**	566/669 **(84%)**		1012/1062 **(95%)**	921/980 **(93%)**
**ICT**	628/894 **(70%)**	905/1006 **(89%)**	1012/1065 **(95%)**		1740/1780 **(97%)**
**Og4C3**	645/902 **(72%)**	880/964 **(91%)**	921/967 **(95%)**	1740/1772 **(98%)**	

Note: Fractions represent the number of negative results for each test (numerator) out of those that were negative by the index test (denominator). The results are of the form: proportion (%). The number of negative index tests (denominator) changes by column because it only includes specimens with valid results by the comparison test (numerator).

**Table 12:** Negative-to-negative test concordance in community survey.

	COMPARISON TEST (Numerator)						
INDEX TEST (Denominator)	BM14	PANLFBC	URSXP	ICT	OG4C3	BLOOD	PCR*
**BM14**		827/880 **(94%)**	825/867 **(95%)**	1394/1520 **(91%)**	1524/1610 **(94%)**	1404/1425 **(98%)**	1585/1592 **(99%)**
**PanLF**	827/1267 **(65%)**		1401/1613 **(86%)**	2376/2544 **(93%)**	2432/2504 **(97%)**	2137/2155 **(99%)**	2512/2525 **(99%)**
**Urine SXP**	825/1344 **(61%)**	1401/1745 **(80%)**		2380/2654 **(89%)**	2296/2470 **(92%)**	1595/1623 **(98%)**	2519/2549 **(98%)**
**ICT**	1394/2178 **(64%)**	2376/3029 **(78%)**	2380/3077 **(77%)**		4903/5089 **(96%)**	3966/3984 **(99%)**	5149/5166 **(99%)**
**Og4C3**	1524/2320 **(65%)**	2432/3053 **(79%)**	2296/2932 **(78%)**	4903/5164 **(94%)**		4051/4062 **(99%)**	5281/5288 **(99%)**
**Blood Smear**	1404/2254 **(62%)**	2137/2873 **(74%)**	1595/2131 **(74%)**	3966/4364 **(90%)**	4051/4372 **(92%)**		4375/4392 **(99%)**
**PCR** *	1585/2574 **(61%)**	2512/3319 **(75%)**	2519/3336 **(75%)**	5149/5660 **(90%)**	5281/5716 **(92%)**	4375/4408 **(99%)**	

Note: Fractions represent the number of negative results for each test (numerator) out of those that were negative by the index test (denominator). The results are of the form: proportion (%). The number of negative index tests (denominator) changes by column because it only includes specimens with valid results by the comparison test (numerator).

*Based on 10 µl blood specimen.

In the community survey, Og4C3 detected 87% and 91% of the blood smear and PCR positive results, respectively, while ICT detected 80% and 78% of the blood smear and PCR positive results, respectively (Table 10). The positive concordance between ICT and Og4C3 ranged from 53% (ICT positives testing positive by Og4C3) to 62% (Og4C3 positives testing positive by ICT). Of the microfilaremic individuals (positive by blood smear) only 61% were positive by a 10 µl PCR. Conversely 75% of PCR positive individuals were also positive by blood smear. Among the antibody tests, Bm14 identified 90% of individuals positive by PanLF or urine SXP.

Negative test concordance in the school survey (Table 11) revealed that 98% of antibody negative individuals (by Bm14 or PanLF) also tested negative by the antigen tests (ICT or Og4C3) (i.e. few people had filarial antigenemia in the absence of a detected antibody response). Bm14 had the poorest negative concordance with the remaining tests in the school surveys; only 66–72% of those specimens negative by PanLF, urine SXP, ICT or Og4C3 were also negative by Bm14. However, since antibody tests are expected to be the most sensitive at detecting exposure to LF, it is possible that specimens negative for antigenemia would still be ‘true positive’ for Bm14 antibody.

The negative concordance of the antigen tests with the antibody tests was somewhat less in the community survey compared to the school survey, with 90–97% of antibody negative specimens (by Bm14 or PanLF) also testing antigen negative (by ICT or Og4C3) (Table 12). The pair-wise specificity of Bm14 was similarly low in the community survey, as compared to the school survey, with Bm14 identifying as negative approximately two thirds of results that were negative by any of the remaining tests. Comparatively PanLF identified as negative 74–94% of results that were negative by the remaining six tests.

In the absence of a true gold standard test for LF infection, operational definitions of positive and negative gold standards were used to calculate sensitivity and specificity. To measure sensitivity, ‘true positives’ were defined as being either blood smear or PCR positive. The sensitivity of the assays therefore relates to the sensitivity for detecting microfilaremic infections, a measure of justifiable interest to the global LF elimination program, since microfilariae are required to transmit infection. It is more difficult to define a gold standard for specificity of assays since it is recognized that exposure alone can convert individuals to positive-antibody status. Consequently, ‘true negatives’ for antibody tests cannot be defined based on the results of the antigen and parasite tests, making it impossible to calculate the specificity for the antibody tests. Specificity of the antigen tests can be assessed if one evaluates the ability of the antigen assays to identify individuals who are amicrofilaremic and have no antibody evidence of infection or exposure to infection. ‘True negatives’ for the antigen tests were therefore defined based on negative blood smear and PCR results (both negative or one negative and the other not assessed) as well as negative results for both Bm14 and PanLF. It is important to note that this was a conservative definition of antigen specificity, as only antibody-negative individuals were eligible to be considered ‘true negatives’ by the antigen tests (see Discussion).

Sensitivity and specificity of test performance was calculated using the best-estimate gold standards as defined above. These calculations were limited to French Polynesia, Ghana, and Haiti due to missing values for Bm14 in the remaining countries. Overall, the ICT test was found to be 76% sensitive at detecting microfilaremic infections and 93% specific at identifying individuals negative for both microfilariae and antifilarial antibody (Table 13). Using the same gold standard estimates, Og4C3 was found to be 87% sensitive and 95% specific. Stratifying the results by country revealed a high degree of variability in these estimates. ICT sensitivity ranged from 61% in Ghana to 79% in Haiti and French Polynesia, while ICT specificity ranged from 89% in Haiti to 94% in Ghana. Similarly, the sensitivity of Og4C3 assays ranged from 72% in Ghana to 93% in French Polynesia, while Og4C3 specificity ranged from 92% in Ghana to 99% in French Polynesia. It is important to note that a portion of the variability is due to the relatively small sample sizes in the country-specific results, caused by the gold standard criteria.

**Table 13:** Sensitivity, specificity, and predictive values for antigen tests.

		ICT		Og4C3	
		% (N)	95% Confidence Interval	% (N)	95% Confidence Interval
**All Countries** a					
	Sensitivity	75.5 (94)	(66.8, 84.2)	87.2 (94)	(80.5, 94.0)
	Specificity	92.5 (1647)	(91.2, 93.7)	94.6 (1647)	(93.5, 95.7)
	Pos. Predictive Value	36.4 (195)	(29.7, 43.2)	48.0 (171)	(40.5, 55.4)
	Neg. Predictive Value	98.5 (1546)	(97.9, 99.1)	99.2 (1570)	(98.8, 99.7)
**French Polynesia**					
	Sensitivity	79.3 (29)	(64.6, 94.1)	93.1 (29)	(83.9, 100.0)
	Specificity	92.3 (517)	(90.0, 94.6)	98.6 (517)	(97.6, 99.6)
	Pos. Predictive Value	36.5 (63)	(24.6, 48.4)	79.4 (34)	(65.8, 93)
	Neg. Predictive Value	98.8 (483)	(97.8, 99.8)	99.6 (512)	(99.1, 100.0)
**Ghana**					
	Sensitivity	61.1 (18)	(38.6, 83.6)	72.2 (18)	(51.5, 92.9)
	Specificity	94.3 (754)	(92.6, 96.0)	91.6 (754)	(89.7, 93.6)
	Pos. Predictive Value	20.4 (54)	(9.6, 31.1)	17.1 (76)	(8.6, 25.6)
	Neg. Predictive Value	99.0 (718)	(98.3, 99.7)	99.3 (696)	(98.7, 99.9)
**Haiti**					
	Sensitivity	78.7 (47)	(67.0, 90.4)	89.4 (47)	(80.5, 98.2)
	Specificity	89.1 (376)	(86.0, 92.3)	94.9 (376)	(92.7, 97.2)
	Pos. Predictive Value	47.4 (78)	(36.4, 58.5)	68.9 (61)	(57.2, 80.5)
	Neg. Predictive Value	97.1 (345)	(95.3, 98.9)	98.6 (362)	(97.4, 99.8)

Definition of antigen test accuracy.

‘True Positive’: Blood Smear or PCR (+).

‘True Negative’: Blood Smear and PCR *not* (+); Bm14 and PanLF *not* (+).

a Includes French Polynesia, Ghana and Haiti only; others excluded due to missing values for Bm14.

The sensitivity of the antibody tests at detecting microfilaremic individuals was 81% for Bm14, 73% for PanLF and 55% for SXP in urine (Table 14). Again, there was significant variability in these estimates at the country level, with Bm14 sensitivity estimates ranging from 50% in Ghana to 92% in French Polynesia. PanLF sensitivity ranged from 50% in Tuvalu to 77% in French Polynesia. Urine SXP sensitivity ranged from 32% in Haiti to 92% in French Polynesia. As with the antigen results, small sample size due to the limited number of microfilaremic individuals, is likely to account for some of the variability in the sensitivity estimates.

**Table 14:** Sensitivity, specificity, and predictive values for antibody tests.

		PanLF		Bm14		Urine SXP	
		Rate	95% Confidence Interval	Rate	95% Confidence Interval	Rate	95% Confidence Interval
**All Countries**							
	Sensitivity	73.2 (82)	(63.5, 82.8)	81.1 (74)	(72.2, 90.0)	54.5 (77)	(43.4, 65.7)
	Neg. Predictive Value	99.1 (2390)	(98.7, 99.5)	98.2 (790)	(97.3, 99.1)	97.7 (1522)	(96.9, 98.5)
**French Polynesia**							
	Sensitivity	76.9 (26)	(60.7, 93.1)	92.3 (26)	(82.1, 100)	92.3 (26)	(82.1, 102.6)
	Neg. Predictive Value	99.1 (675)	(98.4, 99.8)	99.5 (438)	(98.9, 100)	99.6 (539)	(99.1, 100)
**Ghana**							
	Sensitivity	–	–	50.0 (16)	(25.5, 74.5)	–	–
	Neg. Predictive Value	–	–	98.8 (680)	(98.0, 99.6)	–	–
**Haiti**							
	Sensitivity	70.8 (48)	(58.0, 83.7)	75.0 (48)	(62.8, 87.2)	31.9 (47)	(18.6, 45.2)
	Neg. Predictive Value	96.8 (447)	(95.3, 98.5)	96.6 (336)	(94.7, 98.5)	94.2 (554)	(92.3, 96.2)
**Sri Lanka**							
	Sensitivity	66.7 (3)	(13.3, 100)	–	–	–	–
	Neg. Predictive Value	99.9 (684)	(99.6, 100)	–	–	–	–
**Tuvalu**							
	Sensitivity	50.0 (2)	(0, 100)	–	–	50.0 (2)	(0, 100)
	Neg. Predictive Value	99.8 (548)	(99.5, 100)	–	–	99.7 (396)	(99.3, 100)
**Zanzibar**							
	Sensitivity	–	–	–	–	42.9 (7)	(6.2, 79.5)
	Neg. Predictive Value	–	–	–	–	99.3 (565)	(98.6, 100)

Definition of antibody test accuracy.

’True Positive’: Blood Smear or PCR (+).

‘True Negative’: Blood Smear and PCR *not* (+); ICT and Og4C3 *not* (+).

## Discussion

Deciding whether or not to stop MDA will be expensive and laborious for countries because of both the sampling and testing requirements, so the selection of the diagnostic tool to use is of paramount importance. Accuracy, programmatic feasibility, testing requirements, time and cost must all be factored into the evaluation of the potential diagnostic tools [10]. The current study arose in response to this challenge. A summary of the features and performance of the seven diagnostic tests evaluated is presented in the supporting table at the end of this paper (Table S1).

A common theme that emerges from this multi-country study is that the majority of the tests did not perform as well as expected, with regards to both accuracy and reliability. Though this finding is disappointing, it is important to note that the study represents an effectiveness trial, with the majority of the tests being conducted under varying conditions on-site or in field laboratories by local technicians. Though all the technicians were well-schooled, there were differences in adherence to established protocols. Indeed, the lessons learned with respect to test performance in this multi-country setting provide valuable insight and will hopefully lead to future test improvements. Some common areas identified for improvement across many of the tests include the need for thorough training of test-readers and lab technicians, along with simplification of logistical issues related to specimen storage, shipping and linking with test results.

Another important concern identified was the need for improved standardization and rigorous quality control of commercially manufactured tests and kits, a problem noted particularly with variability in the lots of commercial kits measuring Bm14 antibodies (CELISA) and the TropBio Og4C3 antigen test. In addition, with an increasing reliance on laboratory tests for programmatic decision making, there is a critical need to provide laboratories with standard operating procedures and assay controls (e.g., samples for standard curves, positive and negative controls) that can be used across all labs. Both efforts are needed to guarantee that results generated across countries are comparable and can be used to make robust program decisions.

Use of eluted filter paper blood spots rather than fresh serum in this study might have contributed to the sub-optimal performance of the Bm14 and Og4C3 ELISA tests. When this study was planned, all investigators on the project agreed that filter paper blood spots should be used for the ELISA tests. Multiple studies have described the equivalence of the blood spot and serum specimens for use in both the Bm14 and Og4C3 assays [11]–[14], but since this analysis was conducted, other studies have suggested that blood spots on filter paper might not perform as well as serum in the Bm14 ELISA, and there has been a call for additional studies to compare the two methods directly [15]. In the present study, project laboratories found that blood spot eluates sometimes produced variable and often high background OD values in the Bm14 ELISA, so that data from these countries had to be rejected (Table 5).

When evaluating the best diagnostic tool for programmatic decision-making, the advantages of point-of-care tests are appreciable. In this study, the anticipated advantages of lab-based tests (i.e. better sensitivity and specificity) were outweighed by the convenience, comparable accuracy, and ability to standardize more easily the point-of-care tests. Given the challenges experienced with the lab-based tests (see Table S1) a point-of-care test appears to be most preferable for assessments leading to a decision on whether or not to stop MDA.

Taking these aspects into consideration, we conclude that the ICT should be the primary tool recommended now for decision-making about stopping MDAs in areas with *W. bancrofti* infections. As a point-of-care card test, the ICT is relatively inexpensive, requires no laboratory equipment, and can be processed in 10 minutes, very consistent with programmatic use. As an antigen test, a “positive” ICT result is indicative of the presence of adult worms and the potential for ongoing transmission—arguably a more appropriate measure for establishing an end-point for MDA than antifilarial antibodies detecting exposure to infection. Additional research is needed to determine whether antibody tests are more appropriate for post-MDA surveillance.

One concern with the ICT that arose from this study was the potential subjectivity involved in determining whether a weak-looking band indicates a positive or negative test. Fortunately, improvements to training and training materials can be expected to resolve some of this anxiety about the test's use. Indeed, with these improvements, the ICT appears as the diagnostic tool best suited for use even in low-resource settings to determine when the end-point for the MDA phase of the LF elimination program has been reached.

This recommendation for the ICT test is not meant to undervalue the relatively good performance of the Og4C3 test, which was even more accurate than the ICT in identifying microfilaremic individuals in this study. However, as a laboratory-based assay, the Og4C3 test provided some additional challenges, including inconsistent product performance over time and quality control in the testing laboratories. The Og4C3 and other ELISA tests have performed well in research labs; our results and experience with quality control have illustrated the potential problems with translating these tools into an operational setting. The Og4C3 provides a satisfactory diagnostic alternative that may be appropriate in settings with well-equipped laboratories and the ability to adhere to a quality assurance strategy.

### Limitations and Areas of Future Research

The absence of a true gold standard test for LF infection was a major limitation of this analysis. The need to define a best-estimate gold standard from the available tests further limited the analysis since tests used in the definition cannot be assessed by the same definition without entering into a tautology (an issue for both PCR and blood smear). To measure the sensitivity and specificity of the tests it was necessary to use the best-estimate gold standard to define “true positive” and “true negative” results and then limit the analysis to specimens falling within either category. Based on the criteria used, individuals who tested *not positive* by blood smear and PCR but *positive* by Bm14 or PanLF (n = 1737) were excluded from sensitivity and specificity calculations for antigen tests, as they were neither “true positive” nor “true negative”. It is important to note that such results are biologically plausible, as they may be indicative of individuals with increasing, but undetectable antigen levels, or they can represent individuals who are no longer infected but still have residual antifilarial antibodies. It is clear, though, that the definitions used to establish test sensitivity and specificity are imperfect because of the impossibility of defining a true gold standard of infection.

The ROC analysis for determining Bm14 and Og4C3 cut-off levels was also contingent upon the best-estimate criteria. Therefore, any systematic errors resulting in misclassification of the tests used in the best estimate gold standard have the potential to influence this analysis. A sensitivity analysis was run, which evaluated the suspected ICT false positives, as well as false positive and false negative results with PCR and blood smear. The results from the sensitivity analysis indicate that the sensitivity and specificity of the tests, and conclusions drawn from this analysis, to be robust under various scenarios of misclassification (data not shown). For example, if all ICT-positive specimens with an Og4C3 quantitative result of “0” (N = 48) were considered “false positives” and recoded as ICT-negative, the sensitivity and specificity estimates would not change significantly.

Finally, additional sources of error, common across many tests and countries, stemmed from external issues. Logistical constraints and risk of specimen contamination varied by country and is likely to have caused some of the variance in test performance. The possibility of reader error cannot be discounted.

Some of this study's findings were unexpected and warrant future research and analysis. Though the overall prevalence of detection of antigen or antibody was similar for a given target, the distributions of the test results suggest that they are performing differently. Whether or not this difference is due to variability of test performance or to the tests' detecting different sub-populations of positive individuals is hard to determine. For example, the correlation between the ICT and Og4C3 antigen tests was much lower than expected (phi coefficient 0.53); however both tests identified similar overall prevalence of antigenemia. Part of the discordance may be explained by the cut-point selected for the Og4C3 test. Cut-points for Og4C3 were defined such that the only “true positive” specimens were those testing positive for microfilariae (blood smear or PCR). This is likely to have limited our ROC analysis to “strong positive” Og4C3 results (those with higher unit values), as previous studies have found Og4C3 unit values to be positively correlated with MF values [16]–[18]. Whether or not this biased our final cut-point is unclear. However, the poor correlation may also suggest that the ICT and Og4C3 test are capturing different aspects of antigenemia. A more controlled laboratory study would be needed to determine if this were the case.

### Next Steps

The selection of the ICT as the best tool for establishing the MDA stopping criteria is a significant programmatic advance. However, further assessment is needed to develop the appropriate guidelines for country program managers eager to decide if they are ready to stop MDA. The selection of a diagnostic test is the first step, but it is necessary to define a “threshold” of positive results below which a country can safely discontinue its MDA program. With the less-than-perfect sensitivity and specificity of the diagnostic tools, such a threshold should be based on statistical criteria that can account for the level of error in the measurement with a 95% confidence interval [4]. Also integral to this assessment is the method by which the population will be sampled, as both sampling strategy and threshold will influence the sample size and power of the surveys used to determine if the stopping MDA criteria are met. Addressing these issues is the focus of ongoing research efforts.

The global community has already made great progress on the path to elimination of lymphatic filariasis. The selection of the ICT test for defining the end-point of MDA, based on both the present study and earlier observations permits the WHO to develop appropriate guidelines that will allow many countries to move closer to stopping their MDA programs. Future studies to evaluate sampling strategies, ICT-based stopping thresholds, and long-term consequences of the stopping decision will increasingly strengthen the evidence base for the programmatic guidelines targeting LF elimination.

## Supporting Information

Checklist S1 **STARD Checklist.**
(DOC)

Table S1 **A summary of the features and performance of the seven diagnostic tests evaluated.**
(DOC)

Flow Chart S1 **STARD flow chart detailing the method for assessment of antibody diagnostic tests.**
(DOCX)

Flow Chart S2 **STARD flow chart detailing the method for assessment of antigen diagnostic tests.**
(DOCX)

Flow Chart S3 **STARD flow chart detailing the method for assessment of microfilariae diagnostic tests.**
(DOCX)
