# Supplementary material for: A Multicenter Evaluation of Diagnostic Tools to Define Endpoints for Programs to Eliminate Bancroftian Filariasis
Source: PLoS Negl Trop Dis. 2012 Jan 17;6(1):e1479. doi: 10.1371/journal.pntd.0001479 (PMC3260316; doi:10.1371/journal.pntd.0001479)
Supplement: Table S1 — A summary of the features and performance of the seven diagnostic tests evaluated. (DOC) [file pntd.0001479.s002.doc]

Table S1. Summary table of test conclusions.

| **Test Name (Target Detected)** | **Accuracy[[1]](#footnote-2)** | **Administration of test** | **Analysis of results** | **Test-to-test (lot-to-lot) reliability** | **Time & Cost[[2]](#footnote-3)** | **Comments** |
| --- | --- | --- | --- | --- | --- | --- |
| Blood Smear[[3]](#footnote-4)  60 µl three-line  (Microfilariae ) | 75% of PCR* positive results were also positive by blood smear | Night blood collection required in areas with nocturnal periodicity; fixing and staining process must be done correctly to ensure readability of test | Consistent ability to identify MF on slide is crucial to accuracy and reliability of test; |  | Processing time: ~2 days (make slide, dry and stain—rate at which slide read can vary by # of slides & microscopist)  Cost:$0.08 |  |
| Pooled qPCR 10 bloodspots of 10 µl each  (Microfilariae ) | 61% of results testing positive by blood smear were also positive by PCR | Night blood collection required in areas with nocturnal periodicity; lab equipment required; testing requires significant technical training |  | Well established quality control procedures; may require the establishment of regional reference laboratories with rigid quality control | Processing time: 1 day per plate (96 samples) with additional time required to test individual specimens from positive pools.  Cost: $0.5 per person for pooled samples; individual tests cost an additional $5 each | Poor sensitivity of PCR* relative to blood smear likely result of using 10µl blood spots for PCR vs. 60 µl used on blood slide (though use of 60 µl would increase cost and time of test) |
| ICT  (Filarial antigen) | 76% sensitive for detecting microfilaria-positive individuals; 93% specificity | Point-of-care card test easy to apply; no lab equipment needed; one card test needed per person | Issues determining whether a weak-looking band is positive or negative; results not stable after 10 minutes with current test | Ability to identify weak positive results varied by reader—potential for false positive results; | Processing time: 10 minutes  Cost: $3.50 | Improvements to ICT platform by manufacturer and better training materials should resolve concerns with false positive results |
| Og4C3  (Filarial antigen) | 87% sensitive for detecting microfilaria-positive individuals. 95% specificity | Skilled lab technician and lab equipment required to perform ELISA test properly; filter paper spots of whole blood used for test | Unit value cut-off for positive results must be defined[[4]](#footnote-5) | Commercially manufactured kits not adequately standardized; need for experienced diagnostic laboratories with rigid quality control | Processing time: 3 days (from time blood spots arrive in lab)  Cost: $1.25 ($2.50 if run in duplicate) | Greater quality control and kit standardization needed |
| Bm14  (Antifilarial antibody) | Overall greatest proportion of positive results; 81% sensitive for detecting microfilaria-positive individuals. Low negative concordance with other tests[[5]](#footnote-6) | Skilled lab technician and lab equipment required to perform ELISA test properly; filter paper spots of whole blood used for test | Unit cut-off value for positive results must be defined; best cut-off value varied by kit lots | Substantial differences observed in standard curves from different kit lots; Prevalence of positive results ranged from 9% (Ghana) to 53% (Haiti); need for experienced diagnostic laboratories with rigid quality control | Processing time:2 days (once the specimen is in the lab)  Cost:$5.25 (each sample tested in duplicate) | Greater quality control and kit standardization needed; ideally test would be moved to rapid-test platform; |
| PanLF  (Antifilarial antibody) | 73% sensitive for detecting microfilaria-positive individuals. | Point-of-care cassette test; easy to perform; can be performed using whole blood | Problems with un-cleared blood blocking test strip and prohibiting proper reading of results; uncertainty regarding stability of test results after initial reading | Significant lot-to-lot variation, as measured by number of indeterminate results; wide range of ‘indeterminate’ test results (from <1% to >25%) | Processing time: 35 min/specimen; Cost: $2.80 (with WHO discount), else $3.80 | Improvements to test platform by manufacturer and better training should resolve concerns |
| Urine SXP  (Antifilarial antibody) | 55% sensitive for detecting microfilaria-positive individuals. 25% sensitive for detecting children with positive antigen tests | Skilled lab technician and lab equipment required to perform ELISA test; urine had to be preserved with sodium azide in the field, a hazardous substance, in order to prevent bacterial growth |  | Sensitivity of the test varied widely by country (32% - 92%); the urine samples sent for assessment differed considerably by country, with regards to odor and pH, which may have impacted test performance; need for experienced diagnostic laboratories with rigid quality control | Processing time: 3 days (from time that specimen arrives in lab);  Cost: $1.00 (excluding cost of SXP1 recombinant antigen) | More research needed to understand the variation in performance across age groups and countries |

1. This is the overall accuracy, based on results from all five countries combined; in some cases this summary measure might not be the most appropriate due to the high variability by country(see text) [↑](#footnote-ref-2)
2. The costs presented are “per sample” and do not include the cost of equipment or time of technician [↑](#footnote-ref-3)
3. Considered the closest test to gold standard for identifying microfilariae [↑](#footnote-ref-4)
4. Laboratory analysis found the manufacturer-provided cut-off for the unit value that defines a positive result to be likely too high (see text) [↑](#footnote-ref-5)
5. That Bm14 had the lowest negative concordance of all the tests (meaning when other tests are negative, Bm14 is positive) is not necessarily indicative of poorer test performance. Since antibody tests are expected to be the most sensitive at detecting exposure to LF, it is possible that specimens negative for antigenemia would be true positives with respect to the Bm14 antibody test. Therefore, it is possible that the poorer negative concordance is actually an indication of Bm14’s sensitivity. However one cannot rule out the possibility that the test is producing false positive results.

   * Based on 10 mcl blood specimen [↑](#footnote-ref-6)
