# Supplementary material for: A Multicenter Evaluation of Diagnostic Tools to Define Endpoints for Programs to Eliminate Bancroftian Filariasis
Source: PLoS Negl Trop Dis. 2012 Jan 17;6(1):e1479. doi: 10.1371/journal.pntd.0001479 (PMC3260316; doi:10.1371/journal.pntd.0001479)
Supplement: Flow Chart S1 — STARD flow chart detailing the method for assessment of antibody diagnostic tests. (DOCX) [file pntd.0001479.s003.docx]

**Flow Chart S1: Antibody Detection Tests**

PanLF

N=6865

UrSXP

N=4874

Bm14

N=7708

Invalid/ Indeterminate

N=2708

Negative

N=2875

Positive

N=3535

Invalid/ Indeterminate

N=931

Negative

N=3526

Positive

N=1028

Negative

N=3874

Invalid/ Indeterminate

N=0

Positive

N=1000

Eligible

N=8513
