# Supplementary material for: A Multicenter Evaluation of Diagnostic Tools to Define Endpoints for Programs to Eliminate Bancroftian Filariasis
Source: PLoS Negl Trop Dis. 2012 Jan 17;6(1):e1479. doi: 10.1371/journal.pntd.0001479 (PMC3260316; doi:10.1371/journal.pntd.0001479)
Supplement: Flow Chart S2 — STARD flow chart detailing the method for assessment of antigen diagnostic tests. (DOCX) [file pntd.0001479.s004.docx]

**Flow Chart S2: Antigen Detection Tests**

Og4C3

N=8513

Positive

N=438

Negative

N=7342

Invalid/ Indeterminate

N=733

Invalid/ Indeterminate

N=249

Positive

N=688

Negative

N=7361

ICT

N=8298

Eligible

N=8513
