# Supplementary material for: A Multicenter Evaluation of Diagnostic Tools to Define Endpoints for Programs to Eliminate Bancroftian Filariasis
Source: PLoS Negl Trop Dis. 2012 Jan 17;6(1):e1479. doi: 10.1371/journal.pntd.0001479 (PMC3260316; doi:10.1371/journal.pntd.0001479)
Supplement: Flow Chart S3 — STARD flow chart detailing the method for assessment of microfilariae diagnostic tests. (DOCX) [file pntd.0001479.s005.docx]

**Flow Chart S3: Microfilariae Detection Tests**

Eligible

N=8513

Negative

N=5902

Positive

N=93

Negative

N=4641

PCR

N=5980

Blood Smear

N=5686

Positive

N=78
